# Spatial and Temporal Determinants of Anxiety in a Wild Arboreal Primate

**DOI:** 10.1002/ece3.72528

**Published:** 2025-11-18

**Authors:** Edwin J. Parker, Russell A. Hill, Nicola F. Koyama

**Affiliations:** ^1^ School of Biological and Environmental Science Liverpool John Moores University Liverpool UK; ^2^ Primate and Predator Project Medike Nature Reserve Makhado South Africa; ^3^ Department of Anthropology Durham University Durham UK; ^4^ Department of Biological Sciences University of Venda Thohoyandou Venda South Africa

**Keywords:** anxiety, predator‐induced anxiety, scratching, self‐directed behaviour, wild primate

## Abstract

The fear of being eaten can manifest as anxiety in prey species. In captive primates, anxiety‐related behaviours (such as scratching and self‐grooming) typically increase when exposed to a predator model. Despite this, it remains largely unknown whether the perception of predation risk can evoke anxiety, particularly in wild primates. We collected focal observations from adult females from two habituated groups of wild samango monkeys over 12 months to explore whether scratching increased in areas associated with higher perceived predation risk. To validate scratching as an anxiety‐related behaviour, we compared the rates of scratching following an alarm call to a control period. We found scratching occurred significantly more often after an eagle alarm call relative to baseline levels, indicating this may be a reliable anxiety‐related behaviour. We then used a generalised linear mixed model to predict the rate of scratching as a function of perceived predation risk and factors potentially associated with risk. Scratching increased in the summer months and towards the end of the day, but was not influenced by perceived predation risk. Our findings suggest that samangos may adopt other behavioural strategies to mitigate anxiety in ‘high‐risk’ areas, and that anxiety may be reactive, rather than pre‐emptive, in response to predation risk. We propose that scratching may be a useful indicator of reactive anxiety in wild primates and can help to improve knowledge on the environmental factors that induce acute stress in wild populations.

## Graphical abstract

In prey species, encountering a predator, or the mere anticipation of an encounter, can be anxiety‐inducing. This apprehension can manifest as behavioural changes in the short term, but has not been explored extensively in wild species. Our study shows that self‐scratching is a reliable indicator of anxiety in a wild primate, and that areas of reduced vegetation and periods in the late afternoon are also associated with an increase in anxiety.

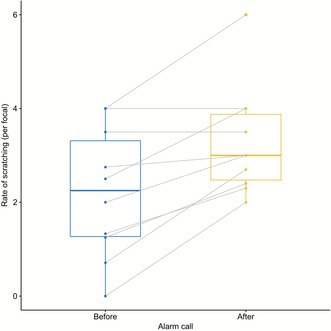

## 1 Introduction

Fear is an adaptive emotional response that serves to modify an individual's behaviour in order to avoid potentially dangerous situations (Nelson et al. 2003). Fear can manifest as anxiety, the apprehension over anticipation of potentially threatening stimuli, which is critical to an individual's survival (Coleman and Pierre 2014). Whilst anxiety serves as a short‐term coping mechanism, prolonged anxiety can lead to chronic stress, which can negatively impact the reproductive success and survival of individuals (Wingfield and Romero 2000; Cox et al. 2010; Crespi et al. 2013). In wild animals, predators are the most significant stimuli invoking anxiety in prey species (Brown 1999), due to the direct, lethal effects of predation. However, the indirect, non‐consumptive effects may be just as significant owing to the influence on individual physiology, population dynamics, and community interactions (Lima 1998; Brown et al. 1999; Laundre et al. 2010; Peers et al. 2018; Wirsing et al. 2021; Parker et al. 2022). As such, even in the immediate absence of predators, prey should maintain a baseline level of anxiety due to the constant possibility of predation (Brown 1999). Yet, predation risk varies across both space and time, such as between safe versus risky habitats or depending on the time of day (Brown 1992, 1999; Brown and Kotler 2004), and so anxiety levels are expected to reflect this variation in risk. Understanding the spatiotemporal variation in anxiety, particularly in the direct absence of predators, can therefore provide important information on the ecological factors that induce anxiety.

Predator‐induced anxiety has been extensively explored using predator models in numerous species (Apfelbach et al. 2005), including rodents (Berton et al. 1998; Belzung and Griebel 2001), zebrafish (Blaser et al. 2010; Maximino et al. 2010), and non‐human primates (Barros et al. 2000). In primates, including humans, long‐term anxiety or stress is typically assessed using faecal glucocorticoids (Rangel‐Negrín et al. 2009; Foerster and Monfort 2010; Crespi et al. 2013; LaBarge et al. 2022). Yet, elevated levels of glucocorticoids do not necessarily mean an individual is anxious or stressed (Beehner and Bergman 2017). Consequently, behavioural indicators of anxiety may be more informative indicators of short‐term, transient anxiety, and are more easily observed in natural environments (Lutz and Baker 2023). Displacement behaviours such as self‐scratching (hereafter, scratching), self‐grooming, body‐shaking, and yawning (Maestripieri et al. 1992; Lutz and Baker 2023), typically referred to as self‐directed behaviours (SDBs), have long been recognised and validated as manifestations of anxiety in nonhuman primates (hereafter, primates) (Baker and Aureli 1997; Kutsukake 2003; Maréchal et al. 2011; Polizzi di Sorrentino et al. 2012; Sclafani et al. 2012; Dell'Anna et al. 2022; Lutz and Baker 2023). Scratching, in particular, was shown to be the most reliable indicator of anxiety in captive chimpanzees (Baker and Aureli 1997) compared to other SDBs. Whilst prolonged anxiety can be detrimental to an individual in terms of psychological stress (Maestripieri et al. 1992), it can also be beneficial in the short term by eliciting the appropriate anti‐predator response, such as increasing vigilance, increasing group cohesion, or evoking predator‐specific alarm calls (Stevenson and Poole 1976), thereby reducing the risk of predation (Lima and Dill 1990; McNamara and Houston 1992; Brown 1999).

Despite some studies citing vigilance as a measure of anxiety (Barros et al. 2000; Coleman and Pierre 2014), vigilant individuals are not necessarily anxious. Studies administering drugs to captive primates have demonstrated the ambiguity of vigilance as an anxiety‐related behaviour. For example, anxiolytics such as lorazepam have been shown to reduce SDBs, such as scratching, in captive cynomolgus macaques ( *Macaca fascicularis* ), whereas anxiogenic compounds increase these behaviours (Schino et al. 1996). In contrast, whilst some anxiogenics have been shown to increase vigilance (Palit et al. 1998), anxiolytics appear to have mixed or negative effects (Schino et al. 1991, 1996; Barros and Tomaz 2002; Barros et al. 2007), suggesting vigilance may represent more general states of arousal (Allan and Hill 2018), Therefore, vigilance alone may not be a reliable indicator of anxiety, and SDBs may be more useful in the context of perceived predation risk.

The majority of work exploring predator‐induced anxiety in primates has thus far focused on captive individuals using predator confrontation tests (Barros et al. 2000, 2004; Barros and Tomaz 2002). These tests presented a taxidermied predator to the study subject in a randomly encountered manner, recording the response of the subject on each occasion (Barros et al. 2000). Responses in marmosets typically involved an increase in alarm calls, vigilance, and SDBs, including self‐grooming and scratching (Barros et al. 2004), although some responses stopped following habituation to the stimulus. Whilst studies on wild primates are lacking, wild‐reared rhesus macaques showed higher levels of anxiety, indicated by behavioural disturbance, when exposed to a snake compared to their laboratory‐reared counterparts (Mineka et al. 1980). However, in both sets of studies, habituation to the stimuli showed marked individual differences, demonstrating the importance of considering individual variation in anxiety (Mineka et al. 1980; Nelson et al. 2003; Barros et al. 2004).

Prey species are known to adopt a variety of anti‐predator behaviours depending on perceived predation risk (Lima and Dill 1990). For example, individuals may avoid areas they perceive to be higher risk (Suhonen 1993; Cowlishaw 1997; Laundré et al. 2001; Heithaus and Dill 2002; Acebes et al. 2013; Coleman and Hill 2014b), alter movement or activity patterns (Fortin et al. 2005; Creel et al. 2008; Valeix et al. 2009; Willems and Hill 2009; Palmer et al. 2017), increase their vigilance (Brown 1999; Campos and Fedigan 2014), or increase the number of nearby individuals (McNamara and Houston 1992; Parker et al. 2022) when accessing high risk areas. Spatial variation in habitat structure also plays an important role in a prey's perceived risk (Cowlishaw 1998; Valeix et al. 2009), as visibility affects the ease of escape of prey species (Lima 1992), whilst aiding predators through camouflage and ambush opportunities (Hopcraft et al. 2005). For arboreal species, height above ground is particularly important, given that areas closer to the ground are viewed as ‘riskier’ (Emerson et al. 2011; Campos and Fedigan 2014; Nowak et al. 2014). Similarly, more open, non‐forested areas are inherently ‘riskier’, due to the increased likelihood of encountering multiple predator guilds, and also increasing the potential for conflict with humans (Nowak et al. 2017; Parker et al. 2022).

Perceived predation risk also varies temporally, resulting from variation in predator/prey activity patterns (Suraci et al. 2022). Prey species may therefore seek to adopt behaviours that minimise risk at certain times of the day and, on a larger scale, across seasons (Suraci et al. 2022). Whilst diel risk may vary depending on the species and habitat in question, sleep site selection has consistently been linked with perceived predation risk in primates (Anderson 1998; Reichard 1998; Day and Elwood 1999; Ramakrishnan and Coss 2001; Fan and Jiang 2008; Albert et al. 2011; Cheyne et al. 2013; Bidner et al. 2018). Anxiety levels may therefore increase when approaching sleep sites owing to the uncertainty over whether sites remain safe on a given day.

Other potential anxiety‐inducing factors include grouping behaviour, which has consistently been cited as an antipredator behaviour in many species (da Silva and Terhune 1988; Fitzgibbon 1990; Magurran 1990; Fortin et al. 2009; Makin et al. 2017; Iranzo et al. 2018), including primates (Hill and Dunbar 1998; Castles et al. 1999; Schmitt and Di Fiore 2015; LaBarge et al. 2020; Dell'Anna et al. 2022; Parker et al. 2022). Thus, more isolated individuals with fewer nearby neighbours may experience increased anxiety (Tkaczynski et al. 2014; Dell'Anna et al. 2022). Furthermore, social stressors, such as competition during mating season (Sclafani et al. 2012), agonistic interactions (Aureli 1997; Castles Duncan and Whiten 1998), the proximity of higher‐ranking conspecifics (Castles et al. 1999), and intergroup encounters (Brooks et al. 2021), may also increase the display of SDBs.

Here, we explored how ‘risk’ perceived by the study species influences scratching rate, an anxiety‐related behaviour, in the arboreal samango monkey ( *Cercopithecus albogularis schwarzi* ), living in a highly fragmented, highly seasonal, and multi‐predator environment (Parker et al. 2020). Predators of the samango monkey include: eagles (African crowned; *Stephanoaetus coronatus* , and Verreaux's; *Aquila verreauxii* ), leopard ( *Panthera pardus* ), and African rock python ( *Python sebae* ). The impact of predation from eagles, in particular, has been shown to be the key driver of samango monkey ranging patterns (Coleman and Hill 2014b), as well as influencing spatial variation in behaviour (Parker et al. 2022). As a result, samangos are expected to display anxiety‐related responses to living in this environment.

To validate scratching as an anxiety‐related behaviour, we predicted that the rate of self‐scratching would increase immediately following a predator encounter, indicated by predator‐specific alarm calls (Fuller 2013), relative to a control period. We predicted that areas of high perceived risk from eagle predation would be associated with an increase in scratching, which may indicate an increase in anxiety associated with these high‐risk areas. Similarly, we also predicted that scratching would increase as individual height above ground decreased, in more open and non‐forested areas, and during periods of low light levels, other factors associated with risk in arboreal primates (Cowlishaw 1998; Hill and Weingrill 2007; Emerson et al. 2011; Cheyne et al. 2013; Campos and Fedigan 2014; Nowak et al. 2014, 2017; Bidner et al. 2018; Parker et al. 2022). Finally, we also assumed monkeys would scratch more in areas of increased intergroup encounter risk, during the hotter, wetter summer months, and in individuals toward the periphery of the group. Individuals should scratch more in areas of increased intergroup encounter, risk due to potential conflict over food resources, territory, and/or mate defence (Lawes and Henzi 1995), and should also increase scratching when becoming increasingly isolated from the group, due to the increased perceived risk associated with increasingly isolated individuals (Tkaczynski et al. 2014; Dell'Anna et al. 2022). Individuals should also scratch more in the warmer, wetter summer months due to the increased need for pelage care and increased ectoparasite load (Ventura et al. 2005).

## 2 Methods

### 2.1 Study Species and Field Site

The samango monkey is an arboreal, diurnal guenon that inhabits the tall‐canopy, evergreen indigenous forests of South Africa (Linden et al. 2016; Wimberger et al. 2017; Parker et al. 2020, 2021, 2022). They live in groups averaging around 40 individuals (Lawes et al. 2013; Coleman and Hill 2014a), and comprising a single adult male, multiple adult females, as well as sub‐adults and juveniles (Henzi and Lawes 1987). The breeding season typically occurs between May and July when other, non‐resident adult males may temporarily form part of the group. Whilst their diet mainly comprises fruits (Lawes et al. 2013; Linden et al. 2016; Nowak et al. 2017; Wimberger et al. 2017; Parker et al. 2020), samangos show considerable dietary flexibility due to living in a highly fragmented and seasonal environment (Nowak et al. 2017; Wimberger et al. 2017; Coleman et al. 2021).

Fieldwork was conducted at the Primate and Predator Project, Lajuma Research Centre (23°02′23″ S, 29°26′05″ E), in the western Soutpansberg Mountains of South Africa. The mountain range experiences warm, wet summers from October to March, and cool, dry winters from April to September. This gives rise to significant diversity in vegetation types (Mostert 2006), including the indigenous mistbelt forest (Mostert 2006; Mucina and Rutherford 2006), which is highly fragmented by montane grasslands, shorter secondary forests (Hahn 2006), residential properties, farmlands, and commercial plantations.

### 2.2 Data Collection

We followed two well‐habituated groups of samango monkeys (‘Barn’, 30–40 individuals, and ‘House’, 60–70 individuals) between August 2016 and August 2017. Five‐minute focal samples (Altmann 1974) were collected using the ‘Prim8’ app (McDonald and Johnson 2014) on a mobile smartphone (Samsung Galaxy S5). Focals were collected on identifiable adult females (through ear tags and/or distinguishing features; ‘Barn’, *n* = 3; ‘House’, *n* = 18) to remove potential age and sex biases in anxiety (Rangel‐Negrín et al. 2009). Groups were followed from morning to evening sleep site (range: 06:00–18:00), with a minimum of 15 min between new focal samples on different, randomly selected individuals. The same individual was not sampled more than twice per day, and only once in the morning and afternoon sessions respectively. Only focal samples lasting the full 5 min were used in analysis, resulting in a total of 249 focal samples over the study period (range 1–22 per individual, *n* individuals: 21, *n* days: 25). Information collected during each focal sample included the date, time, individual ID, group ID, individual height (recorded in m continuously and averaged over the five‐minute sample), the number of conspecific individuals within 5 m (continuous counts averaged across the five‐minute sample), and the number of self‐scratching events (quick, repetitive movements of the hand or foot through fur; Barros et al. 2000). Scratching was recorded as separate bouts when separated by a period of 5 s or more (Castles et al. 1999). We also recorded the individual's location for each focal sample using a handheld GPS (Garmin GPSmap 64S).

To determine perceived predation risk across the home range, we followed the approach of Coleman and Hill (2014b) and Parker et al. (2022). Both the acoustically distinct alarm calls of adult male samango monkeys and, to a lesser extent, group‐wide alarm calls, have been attributed to specific predator guilds (Fuller 2013). In particular, the adult male *ka* and *katrain* calls are associated with raptors (Fuller 2013), whilst group‐wide alarm calls are commonly associated with antipredator behaviour linked to raptors, such as jumping down from the canopy, looking up, and scanning the sky (Fuller 2013). The location of all *ka*, *katrain*, and group‐wide alarm calls was recorded between August 2016 and August 2017, resulting in a total of 356 alarm calls across both groups for the study period. Eighty‐three of these calls were directly associated with a predator (eagle: 74, snake: 9), whilst a further 129 calls were associated with antipredator behaviour from raptors, meaning 60% of alarm calls were directly linked to raptors. We used all alarm calls to create the landscapes of fear, giving an overall spatial map of perceived risk. We also used these alarm calls to validate scratching as an anxiety‐related behaviour in this species.

To determine intergroup encounter risk, the time, location, and details of all encounters were recorded on an all‐occurrence basis. Intergroup encounters were defined as the study group being within visual range of another group, regardless of whether encounters became antagonistic (Coleman and Hill 2014b). There were 104 intergroup encounters recorded across the study period.

We downloaded EVI (Enhanced Vegetation Index) composites from Google Earth Engine (https://earthengine.google.com) from the Landsat 8 databases for the entire western Soutpansberg Mountains at a resolution of 30 m^2^, for each month across the study period (August 2016–August 2017). EVI (Huete et al. 2002) is a remotely sensed measure of plant productivity (Paruelo et al. 1997) and has frequently been used as a proxy for food availability, particularly in species that consume large proportions of leaves (Leimgruber et al. 2001; Ito et al. 2006; Willems et al. 2009).

### 2.3 Data Processing

All data were imported into QGIS 3.0 (QGIS Development Team 2018). To create annual landscape of fear maps for each group, we followed the approach of Parker et al. (2022). Similarly, we used the same approach to create annual maps of group encounter risk, albeit using GPS locations of encounters as opposed to alarm call locations. Monthly EVI composites were selected where cloud cover did not obscure the study area by > 30%, and EVI values for each focal sample location were calculated using the “zonal statistics” plugin in QGIS.

The start time of each focal sample was grouped into one of four categories: early morning (06:00–08:59), late morning (09:00–11:59), early afternoon (12:00–14:59), and late afternoon (15:00–18:00). Grouping time in this way allowed for clearer observation of the effect of time of day on scratching rate. We also grouped focal observations into seasons based on when individuals were sampled (summer, October–March; winter, April–September).

### 2.4 Statistical Analysis

We used the ‘dplyr’ package (Wickham et al. 2017) in R 3.5 (R Core Team 2018) to calculate scratching as a rate per focal sample (frequency per 5 min), as well as the mean number of near neighbours within 5 m for each focal.

To validate scratching as an anxiety‐related behaviour, we compared the scratching rate within 10 min of an eagle alarm call to the scratching rate from focals up to 1 h before the recorded alarm. Rate of scratching before and after an alarm was compared within the same individual (*n* = 10) using a paired *t*‐test with the *t*.test function in R. We used a generalised linear mixed model with a negative binomial error structure to predict the rate of scratching as a function of the landscape of fear, intergroup encounter risk, EVI, season, focal individual height, the number of nearby individuals from the focal individual, and time (with early morning as the reference category). We controlled for individual differences in anxiety by including individual ID as a random effect, in addition to day to control for particular events which may occur on a specific day. Group was included as an additional fixed effect to control for any effects of group size, as well as sampling effort between the two groups. Focal samples where alarm calls occurred during sampling, or in the 30 min prior to sampling, were excluded from analysis (*n* = 4). Models were fitted in R using the glmmTMB function in the ‘glmmTMB’ package (Brooks et al. 2017). We used Akaike's Information Criterion (Burnham and Anderson 2004) to infer goodness‐of‐fit between our model and a null, intercept‐only model. We ran GLMs excluding the random effects and used the VIF (Variance Inflation Factor) function from the ‘car’ package to investigate collinearity between our predictors. We found no evidence of collinearity with all values below 2.0 (Hair et al. 2014). We also found no evidence of spatial autocorrelation after running a Moran's *I* test on the model residuals, using the ‘spdep’ package (Bivand and Wong 2018).

## 3 Results

**FIGURE 1:**
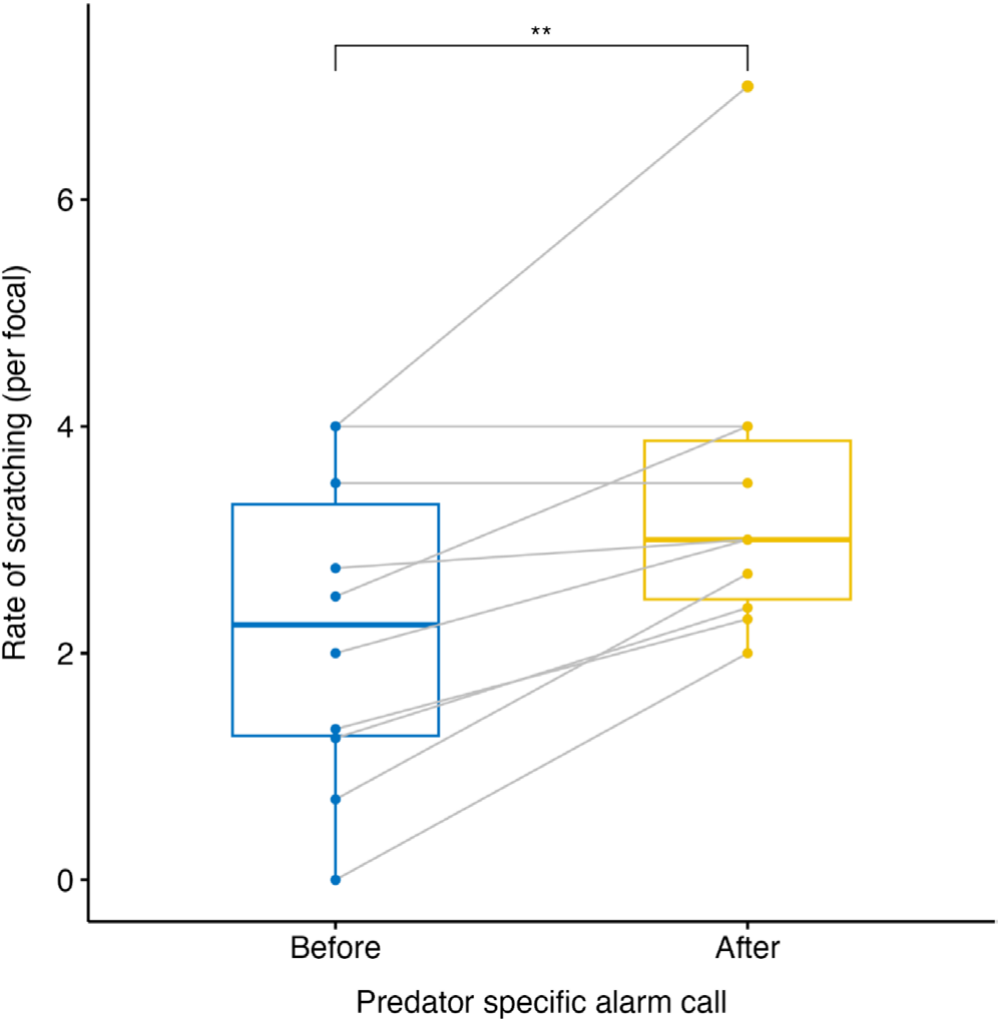
Rate of scratching (average frequency per five‐minute focal observation) up to 1 h before and up to 10 min after an adult male predator‐specific alarm call, in 10 identified adult female samango monkeys at Lajuma Research Centre, Soutpansberg Mountains, South Africa.

Rate of scratching significantly increased in the 10 min after an eagle alarm call relative to the control period (*t*(9) = −4.30, *p* = 0.002, Figure 1). Anxiety was defined as scratching in the subsequent analyses.

Contrary to our predictions, perceived predation risk, indicated by the landscape of fear, did not influence the rate of scratching (Table 1). Similarly, EVI, intergroup encounter risk, individual height, and number of nearest neighbours also had no influence on scratching. We found that the rate of scratching significantly decreased in the winter months, but increased in the late afternoon compared to early morning. The full model, including all predictor variables, performed better than a null, intercept‐only model when comparing AIC values (AIC: 799.5 and 835.0, respectively), suggesting that the variation in the rate of scratching was better explained by behavioural and environmental predictors of anxiety, compared to individual and monthly differences alone.

**TABLE 1:** Coefficient estimates and key statistics of GLMM expressing scratching as a function of landscape of fear, Enhanced Vegetation Index (EVI), season, intergroup encounter risk, individual height, number of nearest neighbors, and time of day.

Coefficient	*B*	SE	*z*	CI_lower_	CI_upper_	Sig
Intercept	0.019	0.354	(1)	−0.675	0.713	(1)
Landscape of fear	0.043	0.079	0.549	−0.111	0.198	0.583
EVI	−0.084	0.077	−1.098	−0.234	0.066	0.272
Season (winter)	−0.416	0.171	−2.435	−0.751	−0.081	0.015
Intergroup encounter risk	0.108	0.081	1.323	−0.052	0.267	0.186
Individual height	0.064	0.075	0.857	−0.083	0.211	0.391
Nearest neighbours	0.106	0.083	1.274	−0.057	0.269	0.203
Time (late morning)	0.082	0.199	0.415	−0.307	0.472	0.678
Time (early afternoon)	0.166	0.203	0.822	−0.231	0.564	0.411
Time (late afternoon)	0.600	0.280	2.141	0.051	1.149	0.032
Group (House)	0.483	0.339	1.426	−0.181	1.147	0.154

*Note:* Nearest neighbours, number of nearby individuals within 5 m of focal subject. (1) not shown because of having no meaningful interpretation.

Abbreviation: EVI, Enhanced Vegetation Index.

## 4 Discussion

In wild animals, the fear of being eaten can evoke anxiety in prey species (Brown 1999). Despite predator‐induced anxiety having been explored extensively in a wide range of species (Hendrie et al. 1996; Barros et al. 2000; Apfelbach et al. 2005; Blaser et al. 2010), studies on primates (Barros and Tomaz 2002), particularly in the wild, are lacking (Mineka et al. 1980). We explored how perceived predation risk and measures of habitat structure associated with risk influenced anxiety‐related behaviour in wild samango monkeys. We found that scratching, a self‐directed behaviour commonly linked to anxiety in primates (Baker and Aureli 1997), significantly increased immediately following alarm calls in response to perceived predation risk from eagles. Nevertheless, spatial perception of risk, indicated by the landscape of fear, did not influence the rate of scratching. Instead, scratching increased during the warm summer months and in the late afternoon.

Self‐directed behaviours, such as scratching, have long been linked with anxiety in captive primates (Baker and Aureli 1997; Barros et al. 2007, 2008). Here, we show that anxiety following the perceived presence of a predator also manifests as scratching in wild samango monkeys. This increase in scratching immediately following an alarm call is unsurprising given the direct costs of mortality from successful predation (Barros et al. 2000, 2004). Anxiety in this case should be beneficial to an individual's survival as it elicits the appropriate antipredator response, such as increasing vigilance and group density, or seeking refuge (Brown et al. 1999).

Interestingly, anxiety‐related behaviours did not increase in areas of high perceived predation risk (indicated by the landscape of fear). However, individuals may pre‐emptively minimise risk (and subsequently anxiety) by adopting antipredator strategies when entering ‘riskier’ areas. Parker et al. (2022) found that groups became more cohesive, reducing distances between conspecifics, when entering areas perceived as ‘high‐risk’ in the same groups of samango monkeys, whilst LaBarge et al. (2020) further found that this response was pre‐emptive. A more cohesive group may seek to confuse predators (Krause and Ruxton 2002; Scott‐Samuel et al. 2015), and/or share the vigilance load between individuals (McNamara and Houston 1992), thereby minimising risk. Consequently, these behaviours may help to minimise the anxiety associated with ‘high‐risk’ areas. Alternatively, anxiety levels in these areas may simply be lowered by the presence of human observers acting as a deterrent towards predators (Emerson et al. 2011; Campos and Fedigan 2014; Nowak et al. 2017; LaBarge et al. 2022). However, the ‘shield’ effect of human observers may be less pronounced in areas of reduced vegetation (where ease of escape is more difficult, regardless of human presence) or when locating sleep sites during low light levels (Cheyne et al. 2013; Bidner et al. 2018). Despite the absence of an effect of perceived predation risk here, the use of vocalisations as an indicator of spatial behaviour is still valuable (Netoskie et al. 2023).

Anxiety in samango monkeys varied temporally, with an increase in scratching rate observed in the late afternoon. This period, particularly the latter part of this period, is consistent with the animals assessing their sleep site locations. Minimising predation risk has long been recognised as a factor influencing sleep site selection in primates (Anderson 1998; Reichard 1998; Day and Elwood 1999; Ramakrishnan and Coss 2001; Fan and Jiang 2008; Albert et al. 2011; Cheyne et al. 2013; Bidner et al. 2018), with arboreal species often preferring the tallest trees in the home range as sleep site locations (Chapman 1990; Barrett and Lowen 1998; Coleman 2013) due to the protection afforded from multiple predator guilds. However, sleep sites that are consistently used can still vary in predation risk owing to the spatial and temporal activity patterns of predators. Consequently, choosing the safest sleep site on a given day is likely to be anxiety‐inducing. Yet, studies exploring this explicitly appear to be lacking. There are, however, examples of primates being exposed to an increased risk of predation when moving towards sleep sites (Reichard 1998; Bidner et al. 2018). Individuals often appear to adopt behaviours that seek to minimise predation risk during this time, such as moving quickly towards sleeping trees (Day and Elwood 1999; Fan and Jiang 2008; Zhou et al. 2009), adopting low‐key, inconspicuous behaviours (Reichard 1998; Day and Elwood 1999; Xiang et al. 2010), and increasing vigilance (Reichard 1998; Zhou et al. 2009; Dayong et al. 2011). A heightened state of anxiety may further prepare individuals for adopting an antipredator response should a predator be encountered, whilst also reflecting the increased risk of locating sleep sites in low light levels when various predators are most active and more difficult to detect (Cheyne et al. 2013; Bidner et al. 2018).

Anxiety was also predicted by season, with self‐directed scratching occurring more often in the hot, wet summer months compared to the cool, dry winters. The summer months at Lajuma are associated with an increase in fruit availability (Coleman 2013), and so it may be reasonable to expect an increase in anxiety during this period due to increased intra‐ and inter‐group aggression. However, samangos show very little within‐group aggression, particularly over resources (Payne et al. 2003), and we similarly found no influence of intergroup encounter risk on anxiety here, thus suggesting fruit availability is unlikely to influence anxiety in samangos. A more likely explanation may therefore be the influence of weather and other associated factors, which correlate with season. In Japanese macaques, self‐scratching was positively correlated with ambient temperature and relative humidity (Ventura et al. 2005). In this instance, scratching likely does not indicate anxiety but rather serves as a hygienic function, with the increased need for pelage care possibly associated with variations in piloerection, sweating, and ectoparasite load (Ventura et al. 2005). Such examples further evidence the multi‐faceted nature of self‐directed behaviours and scratching, in particular, may serve as both a mediator of anxiety and to maintain pelage care. It is important to note that another correlate of anxiety is mating (Sclafani et al. 2012). However, we can rule out any relationship with mating here as the samango monkey breeding season in this subspecies is during the cool, dry winter months when scratching was observed to be lowest.

As forest specialists, samango monkeys are largely restricted to areas of tall‐canopy indigenous forests (Linden et al. 2016; Wimberger et al. 2017; Parker et al. 2020, 2021). Despite this, movement into areas of reduced vegetation cover (indicated by the EVI) did not appear to evoke anxiety. However, samangos have frequently been shown to utilise matrix habitat (Nowak et al. 2017; Wimberger et al. 2017; Parker et al. 2021), and move between forest patches to access specific food resources (Beeson et al. 1996; Coleman and Hill 2014a; Parker et al. 2020). Although inherently riskier for arboreal species, this behavioural flexibility may explain why more open areas with reduced vegetation do not evoke anxiety in this species.

One of the most common stressors in primates is that of fellow group members (Cowlishaw 1997; Hill and Weingrill 2007). Both conflicts between (Dunbar 1988), and the dominance rank (Castles Duncan and Whiten 1998; Castles et al. 1999), of nearby individuals can increase anxiety in the focal individual. In addition, individuals at the periphery of a group may experience more anxiety due to the increased perceived risk of being more isolated (Tkaczynski et al. 2014; Dell'Anna et al. 2022). Whilst we found no effect of the number of nearby individuals on anxiety, the low level of aggression in this species likely serves to reduce anxiety resulting from conspecifics (Lawes 1990; Payne et al. 2003).

More generally, our findings reveal the importance of abiotic factors in influencing anxiety in this species. Both time of day (which could be linked to light levels and temperature), and season (temperature, precipitation and humidity), were the most significant predictors of anxiety in wild samango monkeys. Environmental stress has long been studied in many species, including primates (Kamilar and Beaudrot 2025), yet its impacts on pre‐emptive anxiety are less well‐known. This represents an exciting area for future research, particularly in wild populations, and has implications for behavioural ecology and, on a larger scale, population dynamics, distribution, and evolution (Kamilar and Beaudrot 2025).

Due to the difficulties in identifying and collecting data on arboreal primates in dense forests, particularly guenons (McGraw et al. 2007), we acknowledge certain limitations of our study. Firstly, the findings of our study are based on data collected only on adult females. Whilst this removes any bias relating to age‐sex class and should be representative of a female‐dominated species (Henzi and Lawes 1987), our findings may not reflect the anxiety responses of other species with different social structures. Furthermore, the validation of scratching as an indicator of anxiety is based on data from only 10 adult females. Similarly, caution should be taken when applying SDBs as an indicator of anxiety more generally, and future research should validate our findings further in the context of wild populations. Finally, it is widely acknowledged that the presence of human observers can influence the risk perception of study individuals owing to the “shield” effect (Nowak et al. 2014; LaBarge et al. 2022). Whilst it was beyond the logistical feasibility of our study here, future studies may use remote video monitoring or habituation controls to help mitigate observer‐induced alterations in behaviour.

In conclusion, we show that anxiety in response to perceived predation risk appears to be reactive rather than pre‐emptive in samango monkeys. Scratching rate (a self‐directed behaviour) significantly increased immediately following a predator‐specific alarm call, thus further validating this behaviour as a proxy for anxiety. We also found that anxiety increased during the summer months and in the late afternoon. We suggest that scratching can be a useful indicator of anxiety in wild primates and can help to improve knowledge of the environmental factors that induce stress in wild populations.

## Author Contributions

**Edwin J. Parker:** conceptualization (equal), data curation (lead), formal analysis (lead), investigation (lead), methodology (equal), visualization (equal), writing – original draft (lead). **Russell A. Hill:** conceptualization (equal), formal analysis (equal), funding acquisition (equal), methodology (equal), project administration (equal), resources (equal), supervision (equal), visualization (equal), writing – original draft (equal). **Nicola F. Koyama:** conceptualization (equal), formal analysis (equal), funding acquisition (equal), methodology (equal), project administration (equal), resources (equal), supervision (equal), visualization (equal), writing – original draft (equal).

## Ethics Statement

All behavioural data collection followed the Association for the Study of Animal Behaviour (ASAB) Guidelines for the Treatment of Animals in Behavioural Research and Teaching (ASAB 2012) and were covered by Liverpool John Moores University's use of Live Animals in Unregulated Research Protocol (NK_EP/2016‐10). All fieldwork was approved by the Life Sciences Ethical Review Process Committee and the Department of Anthropology Ethics Committee at Durham University, UK and was conducted with approved permits from the Limpopo Province Department of Economic Development and Tourism (LEDET). It was not possible to record data blind because our study involved focal animals in the field. No human subjects were involved in this study.

## Conflicts of Interest

The authors declare no conflicts of interest.

## Data Availability

The datasets generated and/or analysed during the current study are available in the figshare repository at: https://doi.org/10.6084/m9.figshare.24243430.v1.
